# Liquid Chromatography–Mass Spectrometry-Based Metabolomics Reveals Dynamic Metabolite Changes during Early Postmortem Aging of Donkey Meat

**DOI:** 10.3390/foods13101466

**Published:** 2024-05-09

**Authors:** Wenqiong Chai, Liyuan Wang, Tong Li, Tianqi Wang, Xinrui Wang, Miao Yan, Mingxia Zhu, Jingrong Gao, Changfa Wang, Qiugang Ma, Honglei Qu

**Affiliations:** 1 Liaocheng Research Institute of Donkey High-Efficiency Breeding and Ecological Feeding, Liaocheng University, Liaocheng 252000, China; wanglyuan2023@163.com (L.W.); litong620102@163.com (T.L.); qitianwang9797@163.com (T.W.); wangxinrui0820@163.com (X.W.); 15265087606@163.com (M.Y.); zhumingxia@lcu.edu.cn (M.Z.); wangcf1967@163.com (C.W.); 2 State Key Laboratory of Animal Nutrition, College of Animal Science and Technology, China Agricultural University, Beijing 100193, China; maqiugang@cau.edu.cn; 3 School of Food and Pharmacy, Zhejiang Ocean University, Zhoushan 316000, China; gaojingrong2116@sina.com

**Keywords:** metabolomics, postmortem aging, meat traits, donkey

## Abstract

Background: Metabolic changes in donkey meat during the early postmortem period have not been previously reported. Methods: The LC–MS-based metabolomics technique was conducted to understand the metabolic profiles and identify the key metabolites of donkey meat in the first 48 h postmortem. Results: The pH values showed a decreasing trend followed by an increasing trend. Shear force was the lowest at 4 h and the highest at 24 h (*p* < 0.05). For the metabolome, some candidate biomarker metabolites were identified, such as adenine, inosine, n-acetylhistidine, citric acid, isocitrate, and malic acid. Predominant metabolic pathways, such as citrate cycle (TCA cycle), alanine, aspartate and glutamate metabolism, and purine metabolism, were affected by aging time. Overabundant n-acetylhistidine was identified in LT, declined at 12 h postmortem aging, and then increased. This may explain the significantly lower pH at 12 h postmortem. Adenine was higher at 4 h postmortem, then declined. Decreased ADP may indicate a fast consumption of ATP and subsequent purine metabolism in donkey meat. Conclusions: The results of this study provided new insights into early postmortem aging of donkey meat quality.

## 1. Introduction

In China, Dezhou donkeys are known for their tall, muscular bodies and pure black coloring. Female donkeys may weigh up to 270 kg, while males can weigh up to 300 kg. Usually, donkeys are slaughtered at age two, and the slaughter rate could be approximately 55% [1]. Donkey meat has been a traditional Chinese local cuisine for hundreds of years, particularly in the Shandong and Hebei Provinces. In recent years, donkey meat has been more and more favored because of its nutritious and tender meat characteristics, and the price of donkey meat has increased continuously [2]. Our former research comparing different parts of donkey meat (semitendinosus, longissimus thoracis, and gluteus maximus muscles) showed that the longissimus thoracis muscle was the most tender [3]. In the beef industry, tender beef is more popular with consumers, and they are willing to pay higher prices for it; hence, tenderness is acknowledged as the most important economic trait of meat [4]. Meat quality traits like pH value, tenderness, and meat color are affected by both pre- and postharvest factors. What should be noted is that postmortem aging remains a critical process that could ameliorate meat tenderness and ultimately improve eating quality [5].

As muscle converts into meat, its biochemical metabolism undergoes dramatic changes [6,7]. Aging affects the color and pH value of meat. The color of aged beef becomes brighter and slightly red as certain proteins break down due to enzyme changes [8,9]. Aging causes protein hydrolysis, which produces different amino acids that may increase pH levels [8,9]. Boakye and Mittal [10] reported the instrumental color parameters of the *longissimus dorsi* muscle was caused by the aging time. The vital qualities of raw meat are largely determined by the first 24 h postmortem [7]. Yu et al. [11] have reported that the protein changes in early postmortem periods were associated with glycolysis/gluconeogenesis, oxidative phosphorylation, fatty acid metabolism, the citric acid cycle, and pyruvate metabolism. Throughout all metabolic processes, central carbon metabolism plays an important role in the regeneration of energy, cofactors, substrate degradation, and the supply of biosynthesis precursors [12].

In complex biological processes, metabolomics is widely used to analyze endogenous metabolites [13,14,15]. By identifying and analyzing different metabolites, metabolic pathways can be identified, metabolic networks can be formed, and the functions of metabolites can be determined [16]. Liquid chromatography–mass spectrometry (LC–MS)-based metabolomics allows for the determination of metabolites and metabolic profiles to determine meat quality changes [17,18]. Therefore, in the present study, the LC–MS-based metabolomics technique was conducted to understand the metabolic profiles and identify the key metabolites of donkey meat in the early postmortem aging period. It will be possible to illustrate the variation in metabolism and investigate the main metabolic pathways associated with the aged meat quality of donkeys. In the donkey meat industry, the aging time has been referred to as beef production; this research is expected to reveal the dynamics of the metabolites of donkey meat, which will provide a basis for the application of early postmortem aging in donkey meat production.

## 2. Materials and Methods

### 2.1. Samples

**Figure 1:**
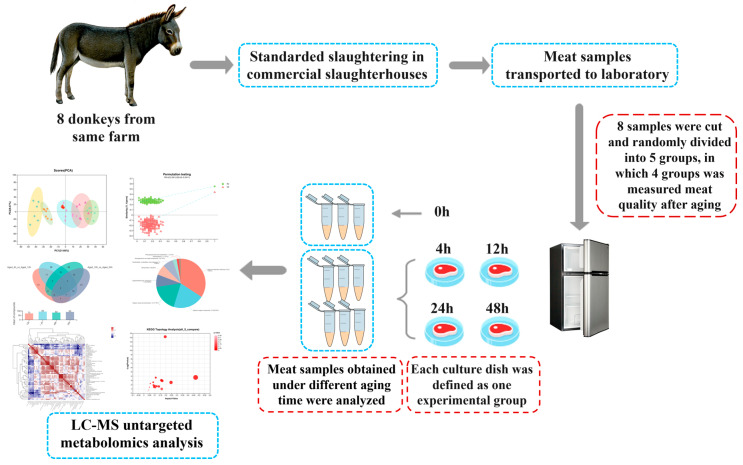
Workflow of this study on the early postmortem aging of donkey meat.

Liaocheng University’s Animal Care and Ethics Committee approved this research (No. 2023042602). Donkeys were slaughtered at a commercial slaughterhouse (Dong’e Tianlong Food Co., Ltd., Liaocheng, China). The Longissimus thoracis (LT) were obtained from donkey carcasses as the samples in this research. Eight male donkeys were selected randomly and slaughtered at the age of 24 months with an ante-mortem weight of 220 ± 15 kg. All the donkeys were transported from the same farm, fasted for 12 h before slaughter, and provided free access to water. The animals were stunned and then bled, and the hides were removed based on commercial practices. The LT muscle from the right side of the carcasses was excised within 30 min postmortem, and the samples were designated as 0 h. The LT muscles were cut into 7 × 7 × 5 cm cubes, placed into a sterile culture dish, and then randomly assigned to one of the following aging times: 4 h, 12 h, 24 h, or 48 h (Figure 1). Aging was performed in a refrigerator at 4 ± 1 °C, without illumination. The cooking loss and shear force were measured after 4 h, 12 h, 24 h, and 48 h of aging. Samples were collected (10 g) from the inside of LT muscles after aging for 0 h, 4 h, 12 h, 24 h, and 48 h. The collected samples were placed in a liquid nitrogen tank immediately and then transferred to a refrigerator at −80 °C for metabolomics analysis.

### 2.2. Meat Quality Analysis

#### 2.2.1. pH Measurement

The pH value of aged donkey LT muscle was measured in triplicate using a Mettler Toledo testo 205 pH metre (Giessen, Germany). The pH meter was calibrated first at pH 6.86 and pH 4.01 at room temperature (approximately 22 ± 2 °C) before measurement. The pH measurement of each sample was conducted immediately after each aging treatment.

#### 2.2.2. Cooking Loss

Cooking loss was measured based on the method by Kim et al. [19], with slight changes. Small chops (2 cm thickness) were obtained from each sample of donkey meat, weighed, and then cooked at 100 °C in a water bath for 5 min. The cooked donkey meat chop was reweighted after cooling for 30 min. The percentage of loss was calculated as the meat sample weight difference.

#### 2.2.3. Shear Force

Shear force was determined using the cooked meat, and three core parallel samples were cut from the cooked meat. Using a TENOVO C-LM3B muscle tenderness meter to measure the shear force (Tenovo International Co., Ltd., Beijing, China), each donkey meat core (approximate 1.5 cm diameter) was sheared at the maximum force, and the average peak shear force (N) was calculated.

### 2.3. LC–MS Untargeted Metabolomics Determination

The LC–MS untargeted metabolomics determination was performed at Majorbio Company (Shanghai Majorbio Bio-pharm Technology Co., Ltd.), Shanghai, China.

#### 2.3.1. Sample Preparation

A total of 40 donkey muscle samples from five aging groups were analyzed for the metabolomics study. A total of 400 μL of extraction solution (1:1 acetonitrile in methanol) was added to 50 mg of each sample. The cells were extracted with ultrasonication for 30 min (5 °C, 40 KHz), followed by centrifugation at 4 °C, 13,000× *g* for 15 min. The supernatant was blown dry with nitrogen and re-solvate with 120 µL of reagent solution (acetonitrile: water, 1:1, *v*/*v*) for 5 min (5 °C, 40 KHz), centrifuged (13,000× *g* 4 °C, 5 min), then transferred to the sample bottle for the next LC–MS analysis. Mixing aliquots into a pooled sample was used to prepare quality control (QC) samples. A repeatability analysis was performed by injecting the pooled sample at regular intervals (every 8 samples) throughout the analytical run.

#### 2.3.2. UHPLC–MS Untargeted Metabolomics Analysis

The analysis was performed using the UHPLC-Q Exactive HF-X platform (Thermo, Ultimate 3000LC). UHPLC–MS conditions and the chromatography parameters were as follows: column, BEH C18 (100 mm × 2.1 mm i.d., 1.8 μm; Waters, Milford, CT, USA); sample injection volume: 2 µL; mobile phase A, water (formic acid containing 0.1%); and mobile phase B, acetonitrile: isopropanol (1:1, *v*/*v*) (formic acid containing 0.1%). A gradient for separation is as follows: 0 to 3 min, mobile phase A decreased from 95% to 80%, increased mobile phase B linearity from 5 to 20%; 3–9 min, A decreased from 80% to 5%, B increased from 20% to 95%; 9–13 min, A and B maintained at 5% and 95%, respectively; 13.0–13.1 min, A increased from 5% to 95%, B decreased from 95% to 5%; and 3.1–16 min, A and B maintained 95% and 5%, respectively. The ion source for MS/MS was 500 °C, the declustering potential was 80 V, the collision energy was 5 V, and the collision energy for MS/MS was 20–60 V.

### 2.4. Data Processing

The raw data were processed using Progenesis QI v3.0 software (WatersCorporation, Milford, CT, USA) for peak identification, retention time correction, and chromatogram alignment. The MS and MS/MS mass spectra were then compared to the metabolic database (http://www.hmdb.ca/, https://metlin.scripps.edu/, accessed on 1 June 2022), with MS mass errors less than 10 ppm, and metabolites were identified using the secondary mass spectra matching scores.

For multivariate statistical analysis, the data matrix was analyzed using the Majorbio Cloud Platform (www.majorbio.com), including a principal component analysis (PCA), partial least squares discrimination analysis (PLS-DA), and variable importance of projection (VIP). Moreover, the difference multiplier and Student’s *t*-tests were performed. OPLS-DA analysis was used to identify metabolites that differ significantly based on their VIP and Student’s *t*-test *p*-values. Significant metabolites were those which had VIP > 1 and *p* < 0.05.

A metabolic pathway annotation was performed through the Kyoto Encyclopedia of Genes and Genomes (KEGG, https://www.kegg.jp/kegg/pathway.html, accessed on 1 June 2022) database to determine the pathways involved in differential metabolites. A KEGG pathway enrichment analysis was conducted using Python’s scipy.stats v1.0.0 package, and Fisher’s exact test was used to find the most relevant biological pathways. *p*-values corrected with Benjamini–Hochberg <0.05 were considered significant.

### 2.5. Statistical Analysis

The meat quality data were analyzed using Minitab version 16; one-way analysis of variance (ANOVA) and Tukey’s test were conducted to determine significant differences between the means. The replication (8 donkeys) and the interaction of replication × postmortem time were not significant. The results are shown as the mean and standard error of the mean (SEM). The significance level was *p* < 0.05.

## 3. Results

### 3.1. Meat Quality

**Figure 2:**
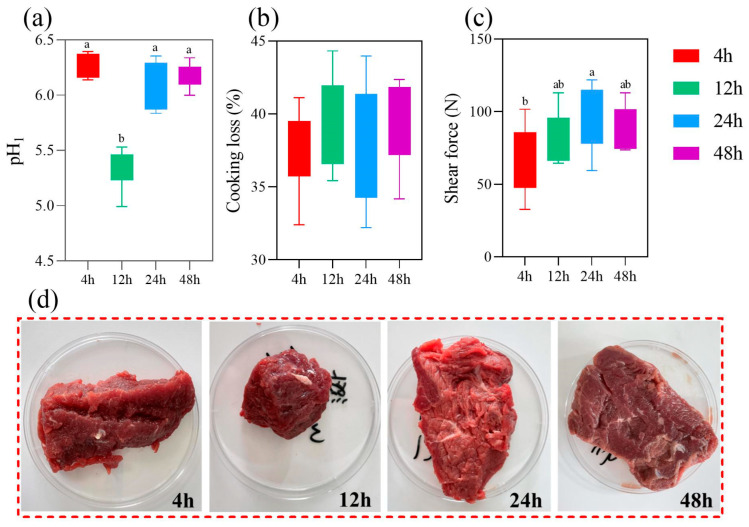
Comparison of quality traits during early postmortem aging of donkey meat. (**a**) pH value. (**b**) Cooking loss. (**c**) Shear force. (**d**) The picture of donkey meat treated with different aging times. Values with different letters (lower a and b) in the bar chat indicate significant differences (*p* < 0.05).

The quality traits of donkey meat in early postmortem aging time are shown in Figure 2a–d. There was no change in cooking loss with postmortem aging (*p* > 0.05). The pH value after aging for 12 h was the lowest compared to 4 h, 24 h, and 48 h (*p* < 0.01). The pH values showed a decreasing trend followed by an increasing trend. Shear force was the lowest after 4 h and highest after 24 h (*p* < 0.05). The results indicated that donkey meat aging for 24 h presents the best appearance.

### 3.2. Metabolomics Analysis

#### 3.2.1. Multivariate Analysis

**Figure 3:**
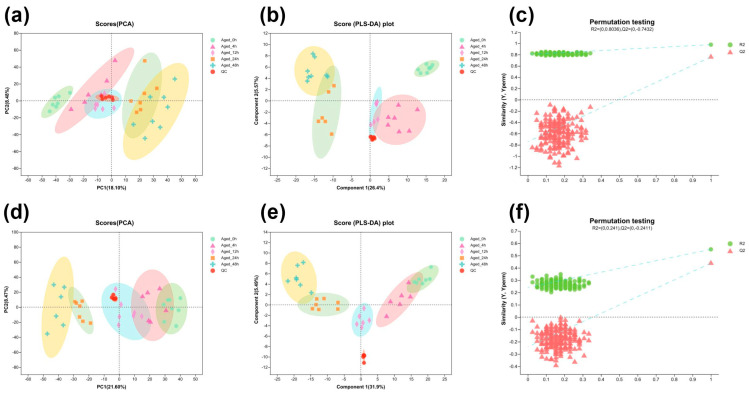
Multivariate statistical analysis of identified metabolites in early postmortem aging. (**a**) PCA score plot of samples acquired in positive mode. (**b**) PLS-DA score plots of samples acquired in positive mode. (**c**) The validation of the PLS-DA model using permutation testing (200 iterations) in positive mode. (**d**) PCA score plots of samples acquired in negative mode. (**e**) PLS-DA score plots of samples acquired in negative mode. (**f**) The validation of the PLS-DA model using permutation testing (200 iterations) in negative mode.

UHPLC–MS detected 6738 peaks, including 3475 positive ion peaks and 3263 negative ions, in samples with different aging times after slaughter. A total of 625 metabolites were identified, including 453 positive ion mode metabolites and 172 negative ion mode metabolites. To discriminate the metabolites among different aging times, a PCA analysis was obtained. The PCA results showed that all the samples were within the 95% confidence interval (Figure 3a,d), as well as the obvious differences among the groups. As the time of aging increased, the differences between 0 h and other hours gradually expanded. The PCA results of 4 h were close to 12 h and 24 h, indicating that the difference in metabolites between them was relatively small. In addition, a PLS-DA model was performed. The PSL-DA model has revealed significant differences between these groups (Figure 3b,e), and the slope of the cross-validated straight line is moderate for 200 permutations, indicating that the PLS-DA model does not exceed the fit in this study (Figure 3c,f), thus indicating that the PLS-DA model was reliable. The results indicated the characteristic differences in metabolites among samples of different aging times. Generally, PCA and PLS-DA multivariate statistical analyses produced highly coincident results.

#### 3.2.2. Qualitative and Quantitative Analysis of Different Aging Time

A summary of the HMDB compound metabolites accumulating with the highest frequency in some of these chemical classifications is shown in Figure S1. Most metabolites were categorized as lipids and lipid-like molecules, organic oxygen compounds, and organic acids and derivatives. The metabolites using VIP > 1 in the OPLS-DA model were screened to identify the different metabolites of samples with the change in aging time. In total, 94, 73, 90, and 83 differential metabolites were identified in the comparison between aging 0 h vs. 4 h, 4 h vs. 12 h, 12 h vs. 24 h, and 24 h vs. 48 h, respectively. In the comparison between 0 h and 4 h, 43 metabolites were upregulated and 51 were downregulated. In the 4 h and 12 h group, 27 and 46 metabolites were upregulated and downregulated. In the 12 h and 24 h comparison, 32 and 58 upregulated and downregulated metabolites were observed, and in the comparison between 24 h and 48 h, 25 metabolites were upregulated and 58 were downregulated.

The Venn diagrams showed the common or unique metabolites among different aging times, and the KEGG chemical classification with all the differential expressed metabolites are shown in Figure S2a,b. To further investigate the correlation of metabolites during the aging period, a cluster heatmap was used to present the importance and expression trends with the differential metabolites. The biological activities of key differential metabolites in each comparison group are shown in Figure S2c,d. The heatmaps were plotted for the compositions of aging 0 h, 4 h, 12 h, and 48 h to present the changes in the top 30 metabolite concentrations.

#### 3.2.3. Enrichment of the Differential Metabolic Pathways

To understand metabolic pathway differences in response to different aging times, an analysis was performed based on the KEGG database. The differential metabolites were mapped in the four comparison groups, and we obtained 36, 13, 27, and 23 differential metabolites with KEGG IDs. The KEGG pathway enrichment analysis was performed, and 22, 3, 13, and 1 pathways with significant difference (*p* < 0.05) diagrams were annotated, respectively (Figure S3). The differential metabolites in the comparison between 0 h and 4 h were mainly annotated in taste transduction, the regulation of lipolysis in adipocytes, purine metabolism, and the phospholipase D signaling pathway, while those in the comparison between 4 h and 12 h were concentrated in the AMPK signaling pathway. In groups 12 h and 24 h, the differential metabolites were concentrated in purine metabolism, lysosome, the phosphatidylinositol signaling system, the glucagon signaling pathway, inositol phosphate metabolism, and oxidative phosphorylation, and in the comparison between 24 h and 48 h, only one pathway identified was significantly enriched (*p* < 0.05), which was purine metabolism. The main metabolites identified were shown in Table 1, and these metabolites may reveal changes in the aging time that affect donkey meat quality.

**Figure 4:**
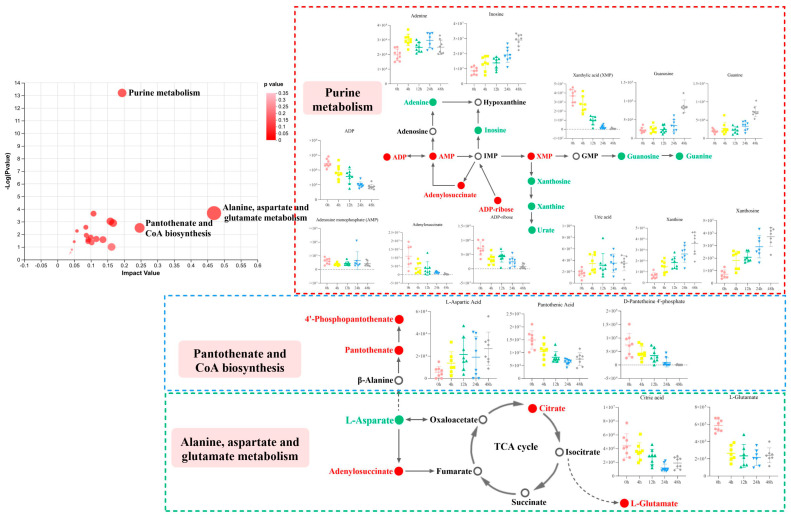
Significantly changed pathways based on the enrichment and topology analysis. The abscissa labels represent five aging times, the red spot represents upregulated metabolites, the green spot represents downregulated metabolites, and the white spot means metabolites were not detected in samples. The colored dashed boxes indicate the levels of significantly up- and down-regulated metabolites in metabolic pathways at different aging times.

The pathways of donkey meat metabolism that affect postmortem aging were determined based on changes in differential endogenous metabolites of donkeys and pathways reported in the KEGG database. These results demonstrate that donkey meat from different aging times reacts to changes in metabolites, which affects its quality. A pathway topology analysis of the different metabolites among the five aging samples was conducted to investigate the most key pathways related to the metabolic response to donkey meat with different aging times. The result can be seen in Figure 4, and the pathway impact and *p* values can be seen in Table 2. Twenty pathways were predicted, and five pathways had impact values greater than 0.15 and *p* < 0.05. These pathways included alanine, aspartate, and glutamate metabolism, pantothenate and CoA biosynthesis, the citrate cycle (TCA cycle), purine metabolism, and caffeine metabolism.

## 4. Discussion

Postmortem aging is an important process in the conversion of muscle to meat. Generally, meat pH affects meat tenderness because it influences proteolytic activity [20]. Polidori et al. [21] have reported that the pH of donkey meat declined and stopped 24 h after slaughtering, but the range of pH value was lower (5.4–5.7) than that of the present study. During the first 48 h post-slaughter, pH decline and its effects on muscle protease activity may contribute to tenderization. It suggested that pH decline is influenced by glycogen content at slaughter, where a lower glycogen content may result in a slower rate of accumulation of glycolysis and lactic acid [22,23]. Hwang and Thompson [24] have reported that an intermediate pH decline (pH 5.9–6.3 at 1.5 h) resulted in tender beef. However, in the present case, the pH of donkey meat dropped dramatically in the first 12 h and then rose, with the ultimate pH value reaching 6.16. The combination of a very rapid pH decline with a slow chilling procedure could cause meat toughness [25]. It is consistent with the results that the pH value of donkey meat declined and the shear force increased within 24 h. The reason could be the earlier depletion of μ-calpain at high temperatures, hence less aging potential during chiller aging [26,27]. Notably, in the present research, the initial pH value was not significantly different than the ultimate pH value. The reason for such a phenomenon may be caused by sampling. Moreover, the slaughterhouse usually rinses carcasses with water several times, which may affect the initial pH value. It is reported that meat pH may be related to the ability of muscle to bind water [28]. The other possible explanation could be free water, which means that during aging, the free water within the muscle cells can be easily mobilized; dehydration creates a region of low water concentration in the surface, resulting in a changed diffusion rate of moisture in the meat, which ultimately affects the pH value [28]. Meat tenderness is a main factor that affects consumer satisfaction. Meat tenderness tends to increase with increasing pH value [28]. Tenderness is also associated with the degradation of the muscle proteins. Moreover, reduced degradation of proteins that tie myofibrils to the cell membrane may result in the shrinkage of muscle cells as a result of shrinking myofibrils [29,30].

Many metabolomics studies have been performed and provided new insights into early postmortem aging [31,32,33]. The process of converting muscle into meat is not only associated with energy-related metabolites but may also affect amino acid and purine metabolism. Based on the metabolomics analysis, several pathways and metabolites were identified in the meat from donkeys in this study, such as the citrate cycle (TCA cycle), pantothenate and CoA biosynthesis, alanine, aspartate and glutamate metabolism, and purine metabolism.

According to previous research, carnosine quantities increased with muscle glycolytic activities [34]. Carnosine is relevant for maintaining the pH homeostasis of muscle cells [35], and higher levels of intramuscular carnosine reveal higher lactate dehydrogenase activity and total buffering capacity in muscle cells [36]. Yu, Tian, Shao, Li, and Dai [31] have reported that N-acetylhistidine and L-histidine were overabundant in *longissimus lumborum*, and higher contents of carnosine and histidine and their H^+^ buffering capacity could lead to an increased pH value. In the present study, n-acetylhistidine and carnosine (β-alanyl-L-histidine) were overabundant in LT; however, the former content declined after 12 h of postmortem aging and then increased. Thus, it could explain the significantly lower pH at 12 h postmortem compared to that of other groups.

Early in the postmortem period, muscle myoglobin stores some oxygen [37], and biological processes in a cell could be sustained by aerobic metabolism for some time because pyruvate is converted into acetyl coenzyme A and then passes to the TCA cycle. According to Yu, Tian, Shao, Li, and Dai [32], the content of metabolites involved in the TCA cycle changed during the postmortem period. Antonelo et al. [38] have reported that the TCA cycle pathway was related to the tenderness of meat, specifically when the metabolites adenine, fumarate, glutamine, and valine have higher concentrations. In the present study, citric acid, isocitrate, and malic acid were numerically overabundant within 4 h postmortem. The result is consistent with Yu, Tian, Shao, Li, and Dai [32], who reported that the content of citrate in *longissimuss lumborum* decreased after 6 h postmortem. Meanwhile, the concentration of adenine was higher at 4 h postmortem and then declined. Those metabolites directly contribute to oxidative metabolism, which forms the substrate for the electron transport chain and ATP synthase [32].

In this study, the contents of inosine and adenine increased with aging time, and ADP was the opposite. Muroya et al. [39] have reported that during postmortem aging, purine metabolism was increased in tender beef. According to Matarneh et al. [40], in the first 24 h postmortem metabolism, the utilization of phosphocreatine is prioritized to maintain a stable concentration of ATP. With the degradation of phosphocreatine, ATP hydrolysis exceeds resynthesis, resulting in excessive ADP formation [40]. Then, ADP goes through purine metabolism, and it produces inosine and hypoxanthine. However, according to Yu, Tian, Shao, Li, and Dai [31], inosine started at a lower value and then increased at 24 h, which was different from the results of the present study. This may indicate that early in the postmortem period, there is fast consumption of ATP and subsequent purine metabolism in donkey meat compared to that of beef. Meanwhile, the accumulation of ADP may also reveal the loss of muscle energy production capability over time. The accumulation of ADP is expected when the rate of ATP hydrolysis exceeds the rate of ATP synthesis [32,41]. Similarly, purine and pyrimidine metabolisms were altered in the postmortem muscle tissue of donkeys based on the identification of other nucleotide degradation metabolites. However, further research is needed to determine whether these changes affect meat color, meat quality, and oxidative stability in postmortem donkey muscle.

## 5. Conclusions

The present study provided a more in-depth view of the metabolomics of donkey meat with aging time. Some candidate biomarker metabolites were adenine, inosine, n-acetylhistidine, citric acid, isocitrate, and malic acid. Predominant metabolic pathways, such as alanine, the citrate cycle (TCA cycle), aspartate and glutamate metabolism, and purine metabolism were affected by aging time. Overabundant n-acetylhistidine declined after 12 h of postmortem aging and then increased; this may explain the significantly lower pH at 12 h postmortem. Adenine was higher at 4 h postmortem and then declined. Decreased ADP may indicate a fast consumption of ATP and subsequent purine metabolism in donkey meat. However, further research needs to validate and quantify that those metabolites could provide critical insight for understanding and improving donkey meat quality.

## Figures and Tables

**Table 1:** Main metabolites of aged donkey meat identified during early postmortem aging.

Group	Metabolite	Formula	VIP	FC	*p*_Value
0–4 h	(R)-S-Lactoylglutathione	C13H21N3O8S	2.4988	1.07	0.0134
	1D-Myo-inositol 3,4-bisphosphate	C6H14O12P2	2.4565	1.09	0.0472
	5-Hydroxyindoleacetic acid	C10H9NO3	1.5848	0.97	0.0084
	5-Hydroxy-L-tryptophan	C11H12N2O3	1.7972	1.05	0.0207
	Acetylcarnitine	C9H17NO4	1.3735	1.02	0.0178
	Adenine	C5H5N5	2.0209	0.96	0.0001
	Adenosine monophosphate	C10H14N5O7P	1.5615	1.03	0.0220
	Adenylosuccinate	C14H18N5O11P	2.8708	1.12	0.0120
	ADP	C10H15N5O10P2	1.4256	1.02	0.0055
	ADP-ribose	C15H23N5O14P2	2.4531	1.06	0.0035
	Alpha-D-Glucose 1,6-bisphosphate	C6H14O12P2	2.3833	1.08	0.0095
	Arbutin	C12H16O7	1.1971	0.98	0.0493
	Biocytin	C16H28N4O4S	2.2409	0.89	0.0230
	Creatine	C4H9N3O2	1.8926	0.98	0.0000
	D-Fructose 2,6-bisphosphate	C6H14O12P2	2.5075	1.11	0.0416
	DG (8:0/15:0/0:0)	C26H50O5	1.0996	1.01	0.0208
	D-myo-Inositol 1,4-bisphosphate	C6H14O12P2	2.6324	1.07	0.0182
	D-Xylose	C5H10O5	1.3285	0.97	0.0410
	Fructose 1,6-bisphosphate	C6H14O12P2	2.4915	1.09	0.0182
	Glutathione, oxidized	C20H32N6O12S2	1.7348	1.02	0.0020
	Inosine	C10H12N4O5	1.6979	0.97	0.0304
	L-Aspartic Acid	C4H7NO4	2.4854	0.90	0.0412
	L-Glutamate	C5H9NO4	2.3490	1.06	0.0014
	LysoSM (d18:1)	C23H50N2O5P+	1.7929	1.03	0.0048
	N-Acetyl-alpha-D-glucosamine 1-phosphate	C8H16NO9P	2.3395	0.93	0.0029
	Pantothenic Acid	C9H17NO5	1.3555	1.03	0.0350
	Phosphocreatine	C4H10N3O5P	2.0266	0.89	0.0471
	Phosphoric acid	H3O4P	1.2767	0.98	0.0320
	Pyrophosphate	H4O7P2	1.3779	0.98	0.0223
	S-Lactoylglutathione	C13H21N3O8S	2.3409	1.07	0.0234
	Uric acid	C5H4N4O3	2.2319	0.94	0.0137
	Xanthine	C5H4N4O2	2.6059	0.94	0.0012
	Xanthosine	C10H12N4O6	2.5790	0.93	0.0095
4–12 h	Adenine	C5H5N5	1.2586	1.02	0.0189
	Alpha-D-Glucose 1,6-bisphosphate	C6H14O12P2	2.9650	1.10	0.0191
	Arbutin	C12H16O7	1.3174	1.02	0.0071
	Calcitriol	C27H44O3	1.9349	0.97	0.0093
	D-Fructose 2,6-bisphosphate	C6H14O12P2	2.9761	1.18	0.0456
	DG (8:0/15:0/0:0)	C26H50O5	1.3920	0.99	0.0071
	Fructose 1,6-bisphosphate	C6H14O12P2	4.4890	1.21	0.0016
	Glutathione, oxidized	C20H32N6O12S2	1.3512	1.02	0.0495
	Glyceric acid	C3H6O4	1.9644	0.95	0.0171
	Oxidized glutathione	C20H32N6O12S2	2.2871	1.04	0.0113
	S-Adenosylhomocysteine	C14H20N6O5S	1.3135	0.98	0.0311
12–24 h	(R)-S-Lactoylglutathione	C13H21N3O8S	3.8322	1.20	0.0258
	1D-Myo-inositol 3,4-bisphosphate	C6H14O12P2	6.5078	3.32	0.0027
	9,10,13-TriHOME	C18H34O5	1.2677	0.97	0.0194
	Acetylcarnitine	C9H17NO4	1.9120	1.03	0.0167
	Adenine	C5H5N5	1.3744	0.97	0.0194
	Adenosine diphosphate ribose	C15H23N5O14P2	5.4947	0.70	0.0001
	ADP	C10H15N5O10P2	1.4856	1.03	0.0101
	Calcitriol	C27H44O3	1.6821	0.97	0.0280
	Cinnavalininate	C14H8N2O6	1.0077	0.99	0.0009
	Citric acid	C6H8O7	2.5784	1.06	0.0018
	Creatine	C4H9N3O2	1.2022	0.99	0.0001
	D-Erythrose 4-phosphate	C4H9O7P	1.9235	1.04	0.0132
	D-Fructose 2,6-bisphosphate	C6H14O12P2	2.8330	1.21	0.0139
	D-Mannose 6-phosphate	C6H13O9P	1.8994	1.04	0.0175
	D-myo-Inositol 1,4-bisphosphate	C6H14O12P2	3.8493	1.20	0.0070
	D-Myoinositol 4-phosphate	C6H13O9P	1.7607	1.03	0.0063
	D-Pantetheine 4’-phosphate	C11H23N2O7PS	3.2471	1.17	0.0191
	D-Xylose	C5H10O5	1.6564	0.95	0.0045
	Glyceric acid	C3H6O4	2.8808	0.91	0.0003
	Inosine	C10H12N4O5	1.1417	0.98	0.0384
	Isocitrate	C6H8O7	2.6414	1.08	0.0003
	Pyrophosphate	H4O7P2	1.2376	0.97	0.0359
	S-Adenosylhomocysteine	C14H20N6O5S	1.5438	0.98	0.0044
	S-Lactoylglutathione	C13H21N3O8S	3.1306	1.19	0.0220
	Uridine diphosphate-N-acetylglucosamine	C17H27N3O17P2	1.7501	1.03	0.0016
	Urocanic acid	C6H6N2O2	1.0355	0.99	0.0028
	Xanthine	C5H4N4O2	1.3893	0.97	0.0339
	Xanthylic acid	C10H13N4O9P	3.7871	1.21	0.0023
24–48 h	Guanosine	C10H13N5O5	3.5774	0.91	0.0003
	Guanine	C5H5N5O	3.3936	0.92	0.0002
	3-Methylindole	C9H9N	1.3661	0.98	0.0229
	LysoSM(d18:1)	C23H50N2O5P+	2.5364	1.04	0.0108
	DG (14:0/16:1(9Z)/0:0)	C33H62O5	1.2358	1.01	0.0144
	S-Adenosylhomocysteine	C14H20N6O5S	1.3707	0.99	0.0085
	(R)-S-Lactoylglutathione	C13H21N3O8S	3.5401	1.16	0.0353
	ADP-ribose	C15H23N5O14P2	3.3305	1.12	0.0313
	D-myo-Inositol 1,4-bisphosphate	C6H14O12P2	3.3973	1.14	0.0240
	Inosine	C10H12N4O5	2.0781	0.97	0.0015
	Theobromine	C7H8N4O2	1.4483	0.98	0.0014
	1,4-beta-D-Glucan	C18H32O18	2.3819	0.96	0.0028
	Glyceric acid	C3H6O4	3.2365	0.93	0.0002
	Biocytin	C16H28N4O4S	2.4494	0.92	0.0439
	KAPA	C9H17NO3	1.9517	0.97	0.0190
	Palmitoyl-L-carnitine	C23H45NO4	2.3837	1.04	0.0491
	9,10,13-TriHOME	C18H34O5	3.0082	0.91	0.0104
	Adenylosuccinate	C14H18N5O11P	3.9232	1.21	0.0035
	Adenosine diphosphate ribose	C15H23N5O14P2	3.3072	0.90	0.0084
	D-Xylose	C5H10O5	2.7604	0.93	0.0000
	Xanthylic acid	C10H13N4O9P	3.4229	1.18	0.0171
	Malic acid	C4H6O5	1.9394	1.03	0.0069

**Table 2:** List of metabolic pathways using enriched analysis for the metabolites.

Pathway Name	Total	Hit	Impact Value	*p* Value
Alanine, aspartate and glutamate metabolism	28	4	0.469565	0.002409
Pantothenate and CoA biosynthesis	27	3	0.246255	0.011784
Purine metabolism	81	13	0.193812	0.000000
Citrate cycle (TCA cycle)	20	3	0.166651	0.006359
Aminoacyl-tRNA biosynthesis	52	2	0.162162	0.138972
Caffeine metabolism	18	3	0.158590	0.005423
Biotin metabolism	23	2	0.136285	0.055364
Amino sugar and nucleotide sugar metabolism	107	4	0.116323	0.054167
Glyoxylate and dicarboxylate metabolism	54	5	0.107896	0.001992
Glycolysis/Gluconeogenesis	31	2	0.102451	0.073197
Fructose and mannose metabolism	52	3	0.099593	0.043905
Arginine biosynthesis	23	2	0.091707	0.055364
Pyruvate metabolism	28	3	0.091410	0.067980
Inositol phosphate metabolism	45	3	0.086398	0.035429
Tryptophan metabolism	56	4	0.084945	0.012193
Histidine metabolism	33	3	0.058318	0.018566
Arginine and proline metabolism	72	3	0.050089	0.067010
Neomycin, kanamycin and gentamicin biosynthesis	76	2	0.042962	0.211768
Glycerolipid metabolism	32	1	0.039099	0.301516
Pentose and glucuronate interconversions	56	1	0.036312	0.354392

## Data Availability

The original contributions presented in the study are included in the article/Supplementary Materials, further inquiries can be directed to the corresponding authors.

## Supplementary Materials

The following supporting information can be downloaded at https://www.mdpi.com/article/10.3390/foods13101466/s1: Figure S1: Pie chart exhibiting the HMDB biochemical categories of the metabolites identified in different aging groups.; Figure S2: Metabolite differences in meat between treatments measured using metabolomics; Figure S3: KEGG pathways that the distinguished metabolites enriched in different aging groups.
