# Supplementary material for: Liquid Chromatography–Mass Spectrometry-Based Metabolomics Reveals Dynamic Metabolite Changes during Early Postmortem Aging of Donkey Meat
Source: Foods. 2024 May 9;13(10):1466. doi: 10.3390/foods13101466 (PMC11119072; doi:10.3390/foods13101466)
Supplement: Supplementary file 1 [file foods-13-01466-s001.zip › foods-2961574-supplementary.pdf]

Supplementary Material

Figure. S1. The pie chart exhibits the HMDB biochemical categories of the metabolites identified in different aging groups. (a) 0h vs 4h, (b) 4h vs 12h, (c) 12h vs 24h, (d) 24h vs 48h.

Figure. S2. The metabolites difference of meat between treatments was measured by metabolomics. (a) The Venn diagram exhibits the number of different metabolites in four comparisons. (b) The pie chart exhibits the KEGG biochemical categories of the metabolites identified. (c) Correlation analysis of metabolites identified in postmortem aging. (d) Heatmap visualization of differential metabolites.

Figure. S3. The KEGG pathways that the distinguished metabolites enriched in different aging groups. (a) 0h vs 4h, (b) 4h vs 12h, (c) 12h vs 24h, (d) 24h vs 48h.
