# Supplementary figures and images for: Liquid Chromatography–Mass Spectrometry-Based Metabolomics Reveals Dynamic Metabolite Changes during Early Postmortem Aging of Donkey Meat
Source: Foods. 2024 May 9;13(10):1466. doi: 10.3390/foods13101466 (PMC11119072; doi:10.3390/foods13101466)

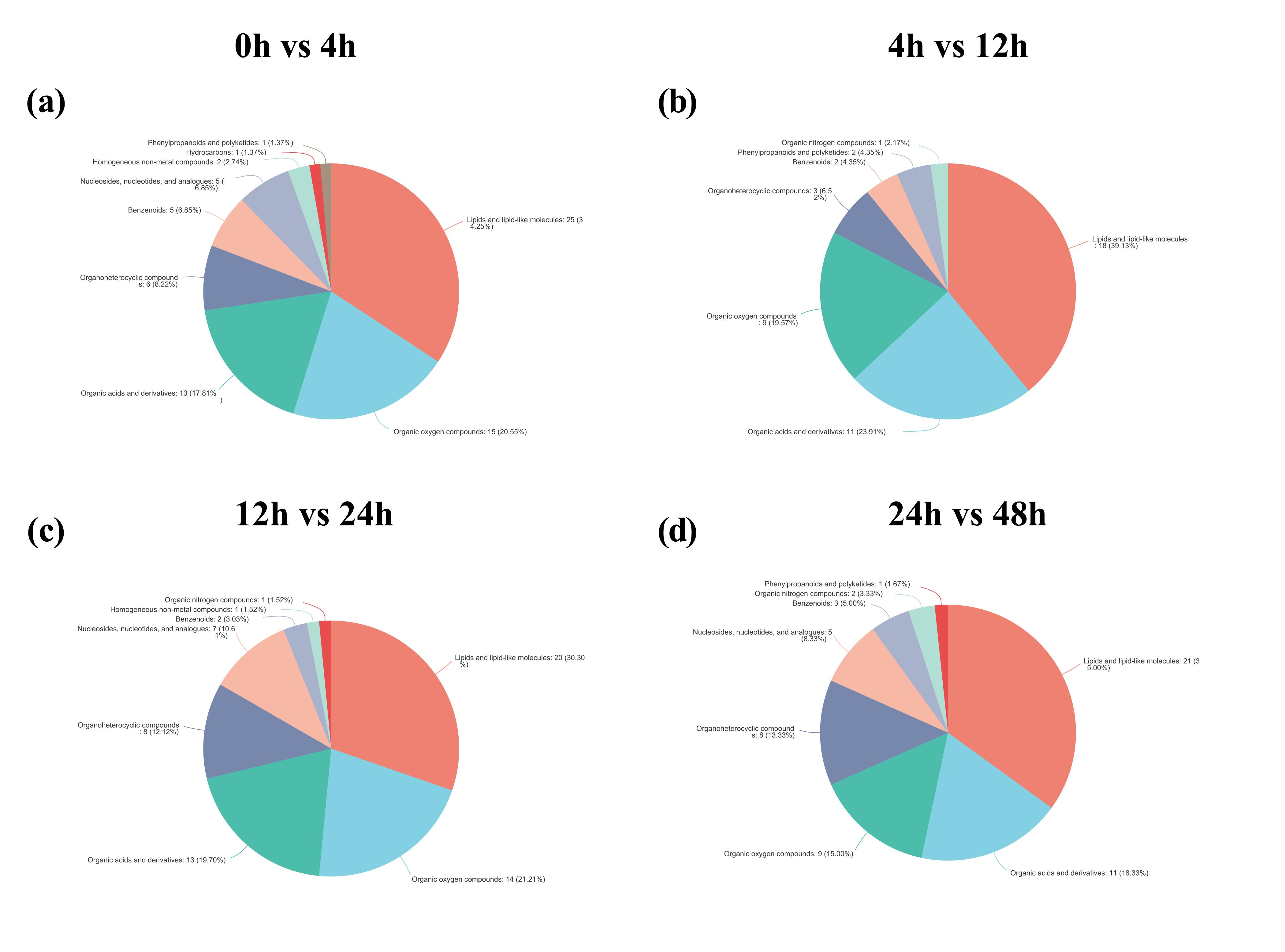

Supplement: Supplementary file 1 [file foods-13-01466-s001.zip › Figure S1.jpg]

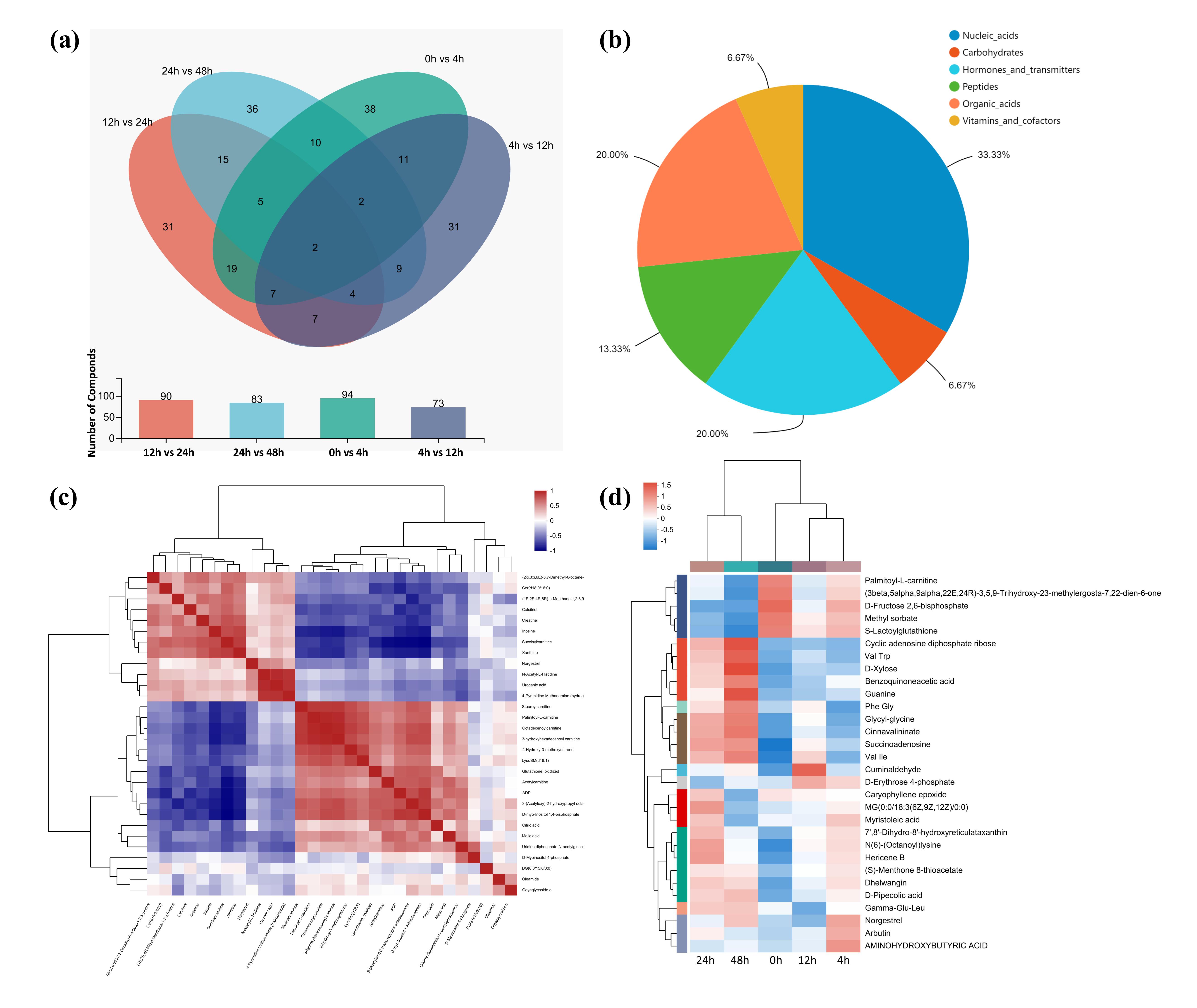

Supplement: Supplementary file 1 [file foods-13-01466-s001.zip › Figure S2.jpg]

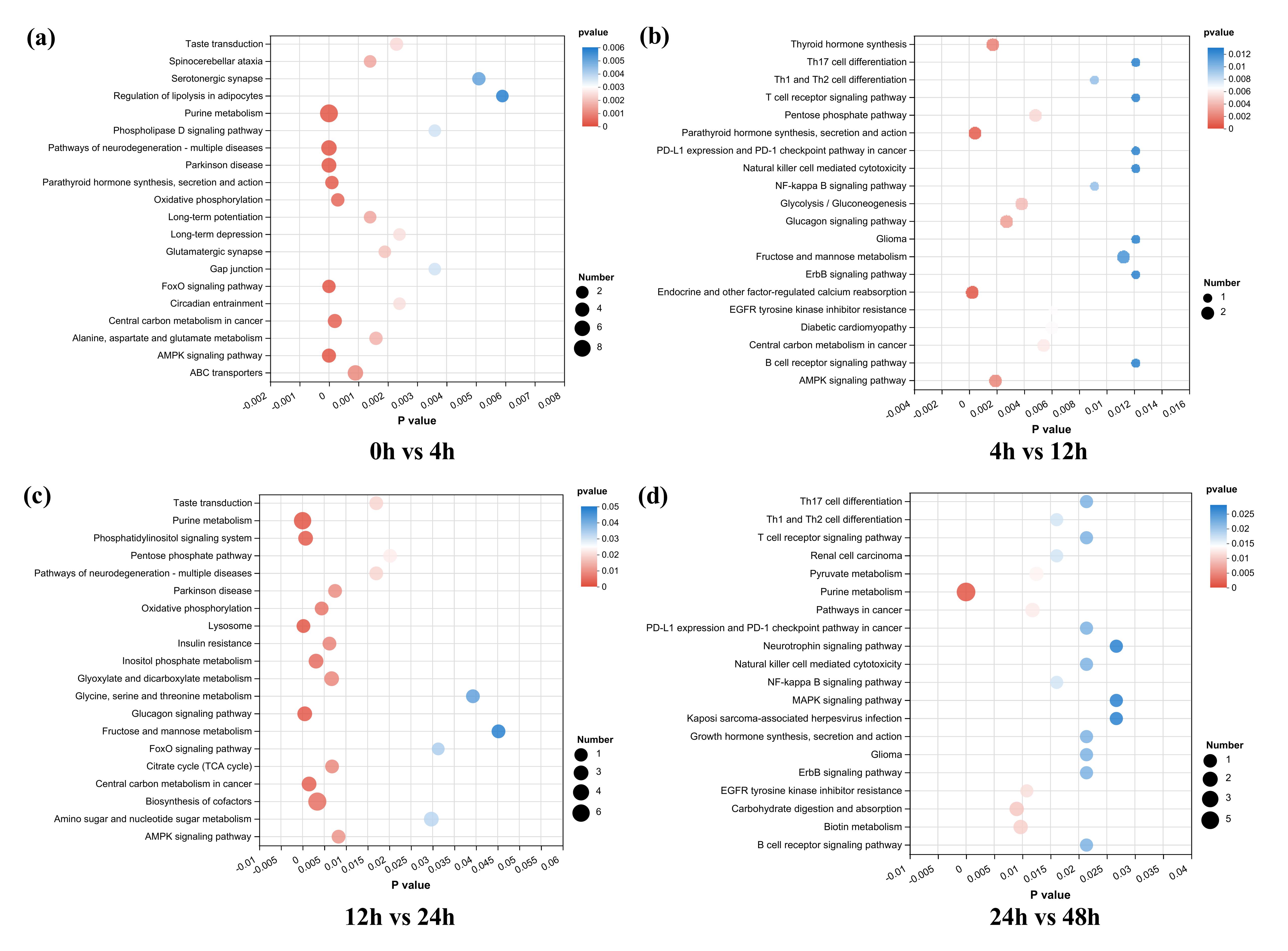

Supplement: Supplementary file 1 [file foods-13-01466-s001.zip › figure S3.jpg]
